# Psychiatrists’ Attitude Towards Smoking Cessation Support (PATSS): Exploring Psychometric Properties of the Measurement Tool

**DOI:** 10.1007/s11126-025-10116-y

**Published:** 2025-01-30

**Authors:** Parul Parul, Bindu Joseph, Sunil Datta, Muhammad Aziz Rahman

**Affiliations:** 1 Institute of Health and Wellbeing, Federation University Australia, 100 Clyde Rd, Berwick Campus, VIC 3806 Australia; 2 The Royal Melbourne Hospital, Melbourne, Australia; 3 School of Nursing, Institute of Health and Management, Level 2/187 Boundary Rd, North Melbourne, VIC 3051 Australia; 4 Collaborative Evaluation and Research Centre (CERC), Federation University Australia, Churchill, Australia; 5 Faculty of Public Health, Universitas Airlangga, Surabaya, Indonesia

**Keywords:** Psychiatrists, Attitude, Smoking, Cessation, Psychometrics

## Abstract

The attitude of psychiatrists plays a crucial role in screening and supporting smoking cessation, especially with people with serious mental illness (SMI). The development of an attitude scale can improve the success of quitting among people with SMI. This study aimed to develop and test the psychometric properties of psychiatrists’ attitudes toward smoking cessation support (PATSS). Based on the literature review, the attitude scale, which comprised 15 items, was developed and tested with 289 psychiatrists. The tool’s psychometric properties were tested by examining item performance, content and construct validity (by exploratory factor analysis (EFA), confirmatory factor analysis (CFA), and reliability. The content validity was demonstrated by content validity ratio (CVR) (0.80) and item content validity index (ICVI) (0.88). Both EFA and CFA identified four factors: Priority and Time Commitment, Recovery Impact and Training, Social Support and Patient Factors, and Coping Mechanisms and Rights. A Cronbach alpha of (0.81) demonstrated high internal consistency. PATSS was found to be a valid and reliable tool to assess the attitude of psychiatrists towards smoking cessation support provided to people with SMI.

## Introduction

Smoking is one of the most common and preventable public health issues globally, killing over eight million people in a year around the world [15]. Smoking among people with mental health disorders is more evident compared to the general population, and they showed lower success in their smoking cessation attempts [39]. There is a high prevalence of smoking among people with serious mental illness (SMI) compared to the general population [3]. Many clinical and population-based studies have identified an association between smoking or nicotine addiction and various mental disorders like depression [36], schizophrenia [22], or bipolar affective disorder (BPAD) [24]. Schizophrenia and related syndromes caused a higher burden of diseases among SMI, leading to higher mortality and morbidity [10].

Psychiatrists are the direct care providers for people with SMI and play a crucial role in assessing, screening, and motivating for smoking cessation. Nevertheless, the delivery or effectiveness of cessation advice relies on psychiatrists’ attitudes towards smoking cessation [14]. Some psychiatrists hesitate to incorporate cessation with primary management care, taking into account the lowered therapeutic efficacy of psychotropic drugs [6], [21] and [37]. Similarly, people with SMI also smoke for various reasons, such as to relieve stress [35], to relieve boredom [17], as a habit [5], or to enhance concentration, or as a coping strategy [27] to manage their emotions. Hence, psychiatrists need to understand the determinants that contribute to smoking and its cessation among people with SMI [3]. Moreover, psychiatrists providing care to people with SMI are not widely aware of their patients’ smoking status or have the presumption that patients are unwilling to quit [19]. Additionally, psychiatrists also experience various barriers, such as time constraints [11], role confusion [18] and lack of training [19].

Exploring psychiatrists’ attitudes toward cessation among people with SMI is imperative to understanding the gap in providing cessation services. The development of a practical attitude scale to assess beliefs, perceived barriers, and enablers related to cessation can inform varied practical interventions and improve the success of quitting among people with mental health issues. There are a range of studies measuring attitudes among staff regarding smoking cessation [4], [26], focussed on the assessing attitudes of psychologists [1], nurse managers [38], and psychiatric nurses [8], [7] but lacked employing a standardised and validated instrument. Another study from Canada used a standardised attitude questionnaire to measure the attitudes of a range of staff related to smoking cessation among people with SMI [16] However, it does not explicitly focus on psychiatrists. Recently, studies have focused on attitudes toward smoking cessation among psychiatric nurses’ [12] in Taiwan and mental health practitioners [29] in Australia, but, to date, there is no published, valid and reliable questionnaires that measure the attitudes of psychiatrists towards smoking cessation amongst people with SMI. Therefore, the study aimed to develop and validate a measure of the attitude of psychiatrists in providing cessation support for smoking among people with SMI.

## Methods

### Study Type and Settings

This methodological study’s analysis included data from another cross-sectional study on the attitudes, self-efficacy, and practice of psychiatrists in providing smoking cessation support among people with SMI in India. Study participants were recruited from the Indian Association of Private Psychiatry (IAPP), a professional organisation in India that represents private and government practitioners in the field of psychiatry.

### Sample and Data Collection

The participants were psychiatrists enrolled in the IAPP, fulfilling inclusion and exclusion criteria. The inclusion criteria were psychiatrists who were practising in psychiatric outpatient departments either in public or private settings, and the exclusion criteria were psychiatrists who completed the online survey in less than 1 min or over 40 min. A Likert scale was used to explore psychiatrists’ attitudes towards smoking cessation. The survey was distributed via the Qualtrics platform to all psychiatrists enrolled in the organisation. The first screen of the online form displayed a plain language information sheet (PLIS) and informed consent, which was then redirected to the second screen, which displayed the survey questions. Only consenting psychiatrists could proceed to the next screen after giving consent. Two subsequent reminders were given to all psychiatrists in a gap of two weeks, and data were collected over two months during 2023. Data were initially collected from 350 participants. However, after applying inclusion and exclusion criteria, 289 responses were received.

### Study Tool Development

The scale was developed after referring to the extensive literature on smoking among people with SMI [23], [30], [32] and [34]. A recent review depicts the correlates of tobacco use among this vulnerable group [25], and other review articles highlighted the need for smoking assessment among people with SMI [9]. Due to the unavailability of a standardised tool for assessing psychiatrists’ attitudes toward smoking cessation among people with SMI, a tool was developed to assess psychiatrists’ attitudes towards smoking cessation. Items of the questionnaire were generated through a deductive-inductive approach. This approach combined a thorough literature review with insights into the dimensional structure of psychiatrists’ attitudes, ensuring that each item was grounded in established research. It included 22 items, scored on a five-point rating scale, with strongly agree (SA) as five and strongly disagree (SD) as 1. The demographic and clinical profiles of participants were also collected.

### Data Analysis

Data were analysed using SPSS (29 Version) and AMOS. Descriptive statistics (frequency and percentage) were used to describe sample characteristics. Variability was analysed in terms of floor and ceiling effects, and a cut-off score of > 15% was used for the minimum and maximum scores for each item. The validity of the scale was assessed for face, content and construct validity. In face validity, the preliminary draft of the attitude scale was evaluated by a panel of eight experts from diverse clinical backgrounds. These specialists assessed each item for its broad applicability and potential ambiguity. Based on their feedback, items were removed to refine the attitude questionnaire’s content. The content validity of the draft was determined, and the attitude scale was distributed to a panel of eight experts (two clinicians, three psychiatrists, and three nurses) with the acquaintance of smoking cessation among individuals with SMI. Each expert evaluated the content validity of each item. Based on their ratings, the Item Content Validity Index (I-CVI) was calculated on a 4-point Likert scale (1 = Not Relevant to 4 = Highly Relevant) to measure relevance and clarity for each item by dividing the number of experts who rated the item as 3 or 4 by the total number of experts. The content validity ratio (CVR) was computed to measure the essentiality of each item on a 4-point Likert scale (1 = Not essential to 4 = essential). Items with an I-CVI value below 0.78 and a CVR value below 0.68 were excluded [2]. Construct validity was assessed through Exploratory factor analysis (EFA), where Kaiser-Meyer Olkin’s measure of sampling adequacy (KMO) and Bartlett’s test of sphericity were used first to determine the suitability of the data for factor analysis with a cut-off was > 0.6 and < 1.0 for KMO, and statistical significance (*p* < 0.001). Confirmatory factor analysis (CFA) was used to examine the adequacy of the resulting factor model. For evaluating model fit, this study used a range of absolute and incremental model fit indices, including the ratio of chi-square to degrees of freedom (X2/df), comparative fit index (CFI), and root mean square error of approximation (RMSEA). Internal consistency reliability was evaluated by Cronbach’s alpha (> 0.7) and item-total correlations (> 0.3).

### Ethical Considerations

Ethics approval was obtained from the Human Research Ethics Committee (HREC) of the Federation University Australia (No: 006/2023) and the Indian Association of Private Psychiatry (IAPP). The researcher collected data through an online Qualtrics survey, where PLIS and consent were displayed on the first screen, and after the agreement, the questionnaire appeared on the second screen to the participants.

## Results

### Participants Characteristics

Table 1 describes the demographic and clinical characteristics of psychiatrists. Just over half of the respondents were males. The majority were non-smokers, and on an average day, they interacted with 20 patients.

**Table 1:** Respondent characteristics

Variable	*N* (%)
Age: Mean (± SD)	39.4 years (± 12.2)
Gender	
Male	156 (52)
Female	146 (48)
Years of clinical working experience	
0–3 years	126 (42)
4–6 years	76 (25)
More than 6 years	100 (33)
Number of patients assessed every day: Mean ± (SD)	20.2 (± 16)
Prior training on smoking cessation	
Yes	136 (45)
No	166 (55)
Smoking status	
Never smoker	219 (73)
Ex-smoker	19 (6)
Occasional smoker	55 (18)
Current smoker	9 (3)

### Data Distribution

The Mean (± SD) score of the attitude scale was 33 ± 8.20, with a median of 33 and a variance of 67.32. The distribution of the total scores had a skewness of 0.50, which is less than one, indicating the normal distribution of data. The kurtosis of the total score came as 0.04, which also indicates a positive normal distribution.

### Data Variability

Variability was assessed by examining the floor and ceiling effect, and > 15% of scores were assigned to the lowest scores across all items, indicating a substantial floor effect. No ceiling effect was observed on any item except for one (the impact on their recovery from psychiatric symptoms). Similarly, all items have floor effects except two items, item − 3 (Impact on their recovery from psychiatric symptoms) and item-15 (Violates the patient’s right to smoke) (refer to Table 2).

**Table 2:** Item performance and reliability estimates of attitude scale (*N* = 289)

Items	Mean	SD	Floor (% with lowest score)	Ceiling (% with the highest score)	Corrected Item-Total Correlation	Cronbach’s Alpha if Item Deleted
I believe smoking cessation among people with SMI :						
Not a priority task	2.60	1.38	24.91	13.15	0.42	0.81
Requires lots of time	1.97	0.97	33.91	2.77	0.44	0.80
Impact on their recovery from psychiatric symptoms	1.97	0.93	12.46	16.61	0.25	0.82
Not on my part of the role	3.23	1.30	23.53	2.08	0.40	0.81
Patient’s initiatives	2.19	0.96	23.53	2.77	0.51	0.80
Likely to impact the therapeutic relationship	2.13	0.96	23.88	2.77	0.43	0.80
Should be delivered following an explicit request from the patient	2.40	1.11	33.91	1.73	0.54	0.80
Depends on organisational support	2.28	1.04	35.29	1.73	0.51	0.80
Requires effective training and skills	1.92	0.89	25.61	1.38	0.48	0.80
Requires patients’ family support	1.73	0.80	42.91	1.38	0.39	0.81
Depends on the patient’s level of motivation	1.69	0.76	44.98	1.04	0.39	0.81
Depends on the patient’s level of self-confidence	1.78	0.84	42.56	1.04	0.40	0.81
Disturbs patients’ coping mechanism	2.20	1.03	27.34	2.08	0.48	0.80
Worsen symptoms of psychiatric illness	2.38	1.11	23.53	3.46	0.46	0.80
Violates the patient’s right to smoke	3.11	1.22	11.07	14.19	0.45	0.80

Total Cronbach’s alpha: 0.81

### Validity

#### Face Validity

Two items were removed to enhance the scale’s clarity and specificity in the assessment of face validity. Therefore, a refined draft comprising 20 items designed to assess psychiatrists’ attitudes was finalised and retained for further testing.

#### Content Validity

For content validity of the attitude scale, I-CVI was calculated for each item, and the mean score of I-CVI was 0.88 after excluding one item (whose score was below 0.78). This conveyed that six out of the eight experts rate an item as “relevant” (usually 3 or 4 on a 4-point relevance scale), and the item would meet the I-CVI threshold for inclusion in the study. The mean score of CVR was 0.80, which means that seven out of the eight experts rated an item as “essential,” and it was considered acceptable, resulting in no further removal of any item. Nineteen items in the attitude scale were drafted and entered into the stage of construct validity.

#### Construct Validity

##### EFA

**Fig. 1:**
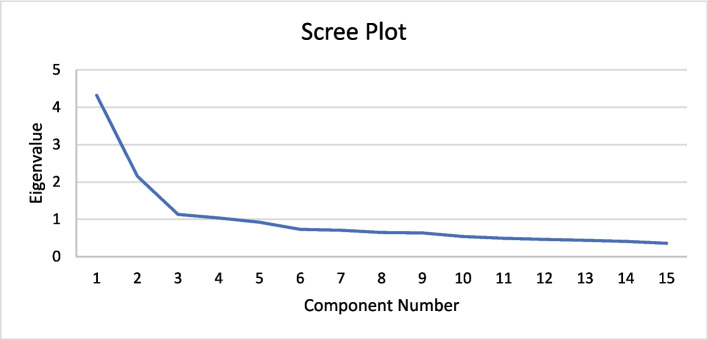
Scree plot

Before conducting EFA, Bartlett’s sphericity test and the KMO measure were performed to assess the suitability of the data for factor analysis. The KMO value was 0.83, indicating a high level of sampling adequacy and supporting the appropriateness of EFA. Additionally, the results of Bartlett’s test (χ2 = 1143.34, df = 105, *p* = 0.001) were statistically significant, confirming sufficient correlations among items for EFA. Based on the criterion that the eigenvalue was greater than 1, 19 items were initiated through EFA. Later, adhering to item selection criteria, four items were eliminated following a stepwise procedure. Item selection was based on a factor load greater than 0.30. In the last round of EFA, 15 items with a factor greater than 1 came up with the four-factor structure (refer to Fig. 1). The cumulative variance was 57.61%, suggesting that the factors were a meaningful capture of data, yielding a reliable conclusion. All items met the criterion of commonalities exceeding 0.30 in the principal component analysis (PCA).

The four factors were named based on the concepts represented by the items within each factor and consulted with experts and the research team: (i) task priority and commitment (5 items) as factor 1, (ii) impacting recovery and training (4 items) as factor 2, (iii) patient factors and support (3 items) as factor 3, and (iv) coping mechanisms and rights (3 items) as factor 4. The pattern loading values obtained from the final attitude scale are presented in Table 3. The standardised factor loading for each item ranged from 0.44 to 0.72 for factor 1, from 0.47 to 0.79 for factor 2, from 0.62 to 0.84 for factor 3, and from 0.53 to 0.69 for factor 4.

**Table 3:** Rotated component matrix

Items	Pattern loading Values	Communalities
***Task Priority and Time Commitment***		
Not a priority task	0.72	0.53
Requires lots of time	0.71	0.64
Not on my part of the role	0.67	0.65
Should be delivered on request of the patient	0.47	0.54
Depends on organisational support	0.44	0.40
***Recovery Impact and Training***		
Impact recovery	0.79	0.61
Patient’s initiatives	0.61	0.51
Likely to impact the therapeutic relationship	0.66	0.51
Requires effective training and skills	0.47	0.49
***Social Support and Patient Factors***		
Requires patients’ family support	0.84	0.57
Depends on the patient level of motivation	0.83	0.67
Depends on the patient level of self-confidence	0.62	0.69
***Coping Mechanisms and Rights***		
Disturbs patients coping mechanism	0.69	0.58
Worsen symptoms of psychiatric illness	0.68	0.63
Violates the patient’s right to smoke	0.53	0.56

Extraction method: Principal component analysis

##### CFA

**Fig. 2:**
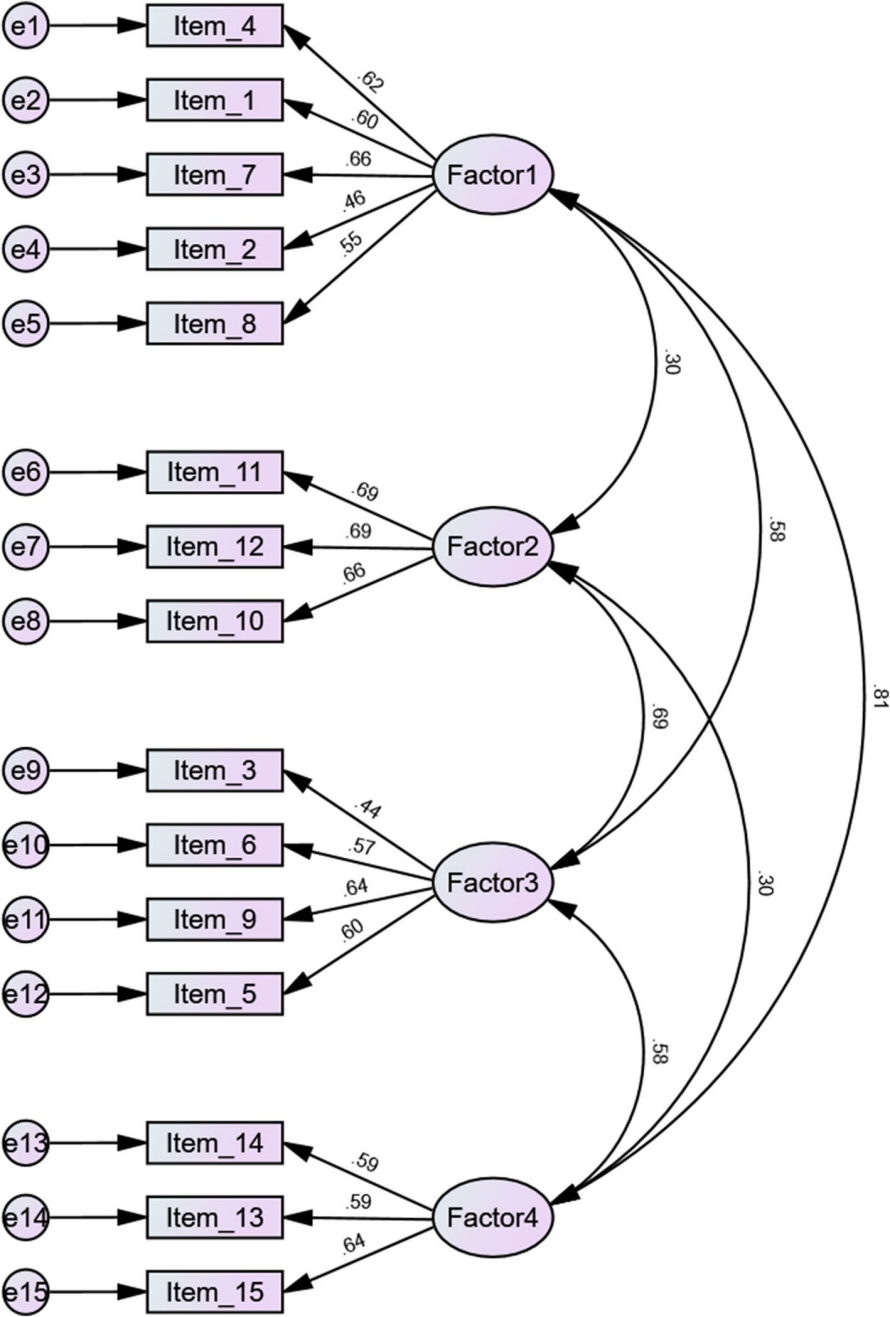
Confirmatory factor analysis of PATSS scale

Post EFA, CFA was performed to examine the adequacy of the resulting factor model. The analysis of the standardisation coefficient of all items was calculated between 0.44 and 0.84 (refer to Figure 2). The values for the fit index model, the RMSEA, SRMR, and the comparative fit index (CFI) were X2/df=2.350, 0.038, 0.075, and 0.902, respectively, which confirmed the optimal fit of the model.

### Reliability

Internal consistency was estimated using a 15-item scale and subscale for psychiatrists’ attitudes. The reliability coefficient of internal consistency for the total scale was 0.81 (refer to Table 2). The Cronbach’s alpha for all the subscales was between 0.80 and 0.82, indicating that the subscales had good internal consistency. While internal consistency has been evaluated to provide an initial measure of the scale’s reliability, test-retest reliability and inter-rater reliability were not assessed.

## Discussion

The current study focused on developing an attitude scale of psychiatrists for smoking cessation among people with SMI. The final version of the attitude scale consisted of 15 items and four subscales. The reliability test (Cronbach alpha) of the current study indicates that the attitude scale has internal consistency, concluding that it is stable [28] and [33]. Furthermore, The validity of the attitude scale was established, and factor analysis confirmed the accuracy of the tool. The results of EFA verified that the tool had multiple sections/ subscales. The four scales after factor analysis were task priority and time commitment, affecting impact and recovery, patient factors, and coping mechanisms. It has been proven that a factor loading of 0.45 was recommended as a threshold [13].

The analysis through KMO and BTS indicated a fit for data factor analysis. The attitude scale identified the psychiatrist’s outlook on smoking cessation among people with SMI. The information extracted from this tool can act as a cornerstone to understanding psychiatrists’ perceived barriers in hindering cessation. This scale can be vital to appraising the psychiatrist’s viewpoint regarding their lack of time, lack of training as well as lack of motivation among patients as some of the significant barriers to smoking cessation [20]. The attitude scale’s significance was that the subscales and items fundamentally originated from qualitative research related to psychiatrist attitude [14] and [31]. Furthermore, this scale presented multidimensional components related to exploring psychiatrists’ attitudes toward cessation. Understanding beliefs and attitudes among psychiatrists plays a crucial role, as it focuses on reflecting the effectiveness of cessation strategies applied for people with SMI.

## Limitations

Despite the rigorous methodology of the study, there were some limitations. Only psychiatrists enrolled in one organisation were included in the study, which may limit the representativeness of the findings. Moreover, the attitudes of the psychiatrists regarding smoking cessation in their clients were collected from the OPD unit only, and these findings may not fully represent the views of psychiatrists in other settings, like inpatient or specialised care units. Also, the absence of test-retest reliability and/or inter-rater reliability assessments restricts the ability to ascertain the temporal stability of the measures and the consistency of findings across different raters or time intervals. These assessments can be considered as future work to enhance the robustness and generalisability of the outcomes under varying conditions.

## Conclusion

The attitude scale was developed to assess the attitudes of psychiatrists in relation to smoking cessation among people with SMI and showed a good level of validity and reliability. Four subscales provided substantial opportunities for targeted training among psychiatrists, leading to improved practices to achieve smoking cessation among people with SMI. In future studies, testing these attitude scales in diverse clinical settings, such as inpatient departments or different countries, may reveal nuances from psychiatrists’ perspectives.

## Data Availability

The datasets used and/or analysed during the current study are available from the corresponding author and study investigators upon reasonable request.
